# Efficacy and Safety of Mirror Therapy for Post-stroke Dysphagia: A Systematic Review and Meta-Analysis

**DOI:** 10.3389/fneur.2022.874994

**Published:** 2022-07-04

**Authors:** Kelin He, Lei Wu, Fengjia Ni, Xinyun Li, Kang Liang, Ruijie Ma

**Affiliations:** ^1^The Third Affiliated Hospital of Zhejiang Chinese Medical University (Zhongshan Hospital of Zhejiang Province), Hangzhou, China; ^2^The Third School of Clinical Medicine (School of Rehabilitation Medicine), Zhejiang Chinese Medical University, Hangzhou, China

**Keywords:** mirror therapy, stroke, dysphagia, meta-analysis, systematic review

## Abstract

**Background:**

Post-stroke dysphagia is a common symptom after stroke and one of the most frequent and severe complications of stroke. Over the recent years, mirror therapy has generated significant research interest as a non-invasive therapeutic and rehabilitative intervention for post-stroke dysphagia and has been investigated in several randomized controlled trials in single center.

**Objective:**

In this study, we aimed to evaluate the efficacy and safety of mirror therapy for post-stroke dysphagia.

**Methods:**

A total of seven databases were searched comprehensively from inception to the 31 December 2021, including PubMed, the Cochrane Library, Web of Science, China National Knowledge Infrastructure (CNKI), Chinese Biomedical Literature Service System (SinoMed), Wan-fang database, and the Chinese Scientific Journals Database (VIP) from inception to 31 December 2021. The primary outcome measure was efficacy, as measured by clinical effectiveness rate. Secondary outcomes included the water swallowing test and the incidence of pneumonia. In addition, we applied the Cochrane Risk of Bias Tool to investigate the risk of bias. Potential publication bias was evaluated by applying Egger's bias indicator test and by assessing the symmetry of data when visualized as funnel plots.

**Results:**

A total of five randomized controlled trials (135 subjects in the experimental group and control group) were found to report the application of mirror therapy for post-stroke dysphagia and were included in this study. No publication bias was detected. Meta-analysis revealed that mirror therapy had a positive effect on the rate of clinical efficacy [odds ratio (OR) = 4.22; 95% confidence interval (CI): 2.3–7.73] and the water swallowing test [mean difference (MD) = −0.76; 95% CI = −1.29 to −0.22]. Moreover, mirror therapy reduced the incidence of pneumonia (OR = 0.13; 95% CI = 0.03–0.49). Subgroup analyses indicated that mirror therapy during the acute phase was robust but was unstable during the convalescent phase. Sensitivity analysis revealed that the results generated by our meta-analysis were robust and stable.

**Conclusions:**

Available evidence appears to suggest that mirror therapy may have a role in the management of post-stroke dysphagia but has yet to be fully confirmed. Existing evidence from clinical trials suggests that evidence relating to the safety of mirror therapy for patients with post-stroke dysphagia is not yet sufficient.

**Systematic Review Registration:**

Identifier: CRD42022302733.

## Introduction

Dysphagia is a common condition and a life-threatening symptom during the acute phase after the onset of stroke; ~80% of patients with stroke experience dysphagia (1). Despite the reduced incidence of dysphagia over time after stroke (2), almost half of all patients continue to experience the symptoms of dysphagia during the convalescence stage (3). Dysphagia can result in a significant increase in the incidence of complications, including aspiration pneumonia, malnutrition, and dehydration, thus leading to a prolonged hospital stay and high medical expenses (4–6). Moreover, post-stroke complications can delay functional recovery and degrade the quality of life when patients cannot eat or drink (7). Therefore, by improving the deglutition function of patients with stroke, we will be able to increase their quality of life.

Mirror therapy, as a non-invasive therapeutic and rehabilitative intervention, has been widely used to improve functional recovery after stroke (8–10). In contrast to other interventions, which tend to employ somatosensory input to assist motor recovery (11), mirror therapy is based on visual stimulation. During the general mirror therapy, a mirror is placed on the patient's midsagittal plane, thus reflecting the non-paretic side as if it were the affected side (12–14). Some authors have recently described “mirror-like” videos or computer graphic systems, in which a video or computer graphic image of the moving limb is presented (15, 16). The main characteristic of mirror therapy is to establish internal behavioral representations and external observations by imitating these events in person (17). In addition, mirror therapy is a relatively simple to administer and represents a convenient the possibility for self-administered home therapy, even for patients with severe motor deficits (18). The neural mechanisms underlying mirror therapy predominantly include the promotion of motor network connections and activation of the primary motor cortex in the brain (19, 20). A previous study indicated that mirror therapy plays a vital role in the recovery of swallowing function (21). Unlike the use of mirror therapy for upper or lower limb functional restraint, the mode of application for mirror therapy when managing dysphagia is to provide visual cues to stimulate swallowing. Consequently, researchers have already investigated the effect of mirror therapy on neuronal activity during swallowing (22). Thus far, mirror therapy has been widely used to improve swallowing function after stroke in China. However, there have been no specific reviews conducted on the specific effect of mirror therapy on dysphagia after stroke. Therefore, there is an obvious and urgent need to determine the efficacy and safety of mirror therapy for post-stroke dysphagia. In this study, we conducted a systematic review and meta-analysis to ascertain the efficacy and safety of mirror therapy for post-stroke dysphagia.

## Methods

### Study Protocol and Registration

This study was based on the Preferred Reporting Items for Systematic Reviews and Meta-Analyses Protocols (PRISMA-P) (23), and the study protocol has been registered on the PROSPERO database (registration number: CRD42022302733).

### Types of Studies

In this study, we only reviewed random control trials (RCTs), although without language or geographical restrictions. We excluded literature reviews, systematic reviews, meta-analyses, prospective cohort studies, retrospective studies, case series articles, and studies with incomplete or missing information.

### Types of Participants

This study only included studies that enrolled participants with dysphagia after stroke. All subjects in those studies were aged above 18 years, and water swallowing test scores ranged from 3 to 5 points. We excluded the participants with dysphagia caused by other diseases.

### Types of Interventions

Patients were treated by a routine therapeutic protocol in the control group, including rehabilitation training or western medicine. In contrast, patients in the experimental group also received mirror therapy, including video or virtual reality options.

### Types of Outcome Measures

The water swallowing test is frequently used in clinical practice as a functional assessment to evaluate swallowing function (24–27). The primary outcome of this study was the clinical effectiveness rate (CER). In brief, the state of efficacy based on the water swallowing test (1–5 points) was classified as either effective or ineffective. No improvement in clinical symptoms (a score in the water swallowing test that was ≥3 points) was considered ineffective. Otherwise, the treatment was considered as effective. The CER was calculated as follows: (total number—ineffective number)/ total number × 100% (28). The secondary outcome measures of this study included the water swallowing test score and the incidence of pneumonia.

### Search Strategy

We conducted a comprehensive search strategy (from inception to the 31 December 2021) in seven electronic databases: PubMed, Cochrane Library, Web of Science, China National Knowledge Infrastructure (CNKI), Chinese Biomedical Literature Service System (SinoMed), Chinese Scientific Journals Database (VIP), and Wan-fang database. Our aim was to retrieve all articles related to RCTs focusing on the use of mirror therapy to treat dysphagia after stroke. The following terms were employed as free-text, keywords, subject words, and medical subject headings (MeSHs): (stroke OR acute stroke OR acute ischemic stroke OR ischemic stroke OR cerebrovascular disorders OR cerebral hemorrhage OR cerebral infarction OR cerebrovascular) AND (mirror movement therapies OR movement therapy OR mirror movement OR mirror therapy OR mirror visual feedback OR wild cards) AND (dysphagia OR swallowing disorder OR deglutition disorders OR oropharyngeal dysphagia OR esophageal dysphagia). There were no restrictions related to country or language. In addition, only published studies were included; we did not search gray literature.

### Study Selection

Totally, two authors (LW and FN) executed the search strategy and downloaded the summaries of relevant publications. All publications were then imported into Endnote software (version X9; Thomson Reuters, Carlsbad, CA, USA) and duplicate publications were removed. Next, two evaluators (XL and KL) checked the publication titles, abstracts, and full texts and independently retrieved the publications that met the inclusion and exclusion criteria. Any discordance between the two evaluators was moderated by a third evaluator (RM).

### Data Extraction

Totally, two authors (XL and KL) independently extracted relevant information from the included trials and imported these data into Microsoft Excel (Version: 2019). We included a wide range of data, including general information (first author, publication year), demographic data (intervention, age, sex, sample size, and stroke onset), and mirror therapy protocol and outcome indices (clinical effectiveness rate, water swallowing test, and the incidence of pneumonia). We contacted the corresponding authors by e-mail if there were ambiguous or insufficient data in the original articles.

### Assessment of Bias Risk in the Included Studies

Totally, two independent reviewers (XL and KL) evaluated the risk of bias for each of the included studies by applying the Cochrane risk of bias assessment tool (29). A total of seven domains were separately evaluated: random sequence generation, allocation concealment, the blinding of participants and personnel, the blinding of outcome assessment, incomplete outcome data, selective reporting, and other important sources of bias. Each domain within the original study was categorized as high, low, or unclear risk. Any discordance between evaluators was discussed with a third author (RM).

### Statistical Analysis

All statistical analyses were performed using R software (https://www.r-project.org/, version 3.6.3). Continuous and binary data were calculated as mean differences (MDs) and odds ratios (ORs). All estimations were presented with 95% confidence intervals (CIs). The random or fixed effects model is based on clinical heterogeneity and methodological heterogeneity among the studies pooled in a meta-analysis (30). The sources of statistical heterogeneity between studies were tested by *I*^2^ and *Q*-test statistics; a value below 50% represented lower levels of heterogeneity (31). Furthermore, we performed subgroup analysis according to stroke phase and sensitivity analysis by deleting studies on a one-by-one basis. Potential publication bias was evaluated by Egger's bias indicator test and visualized by funnel plot asymmetry (32).

## Results

### Literature Selection

**Figure 1:**
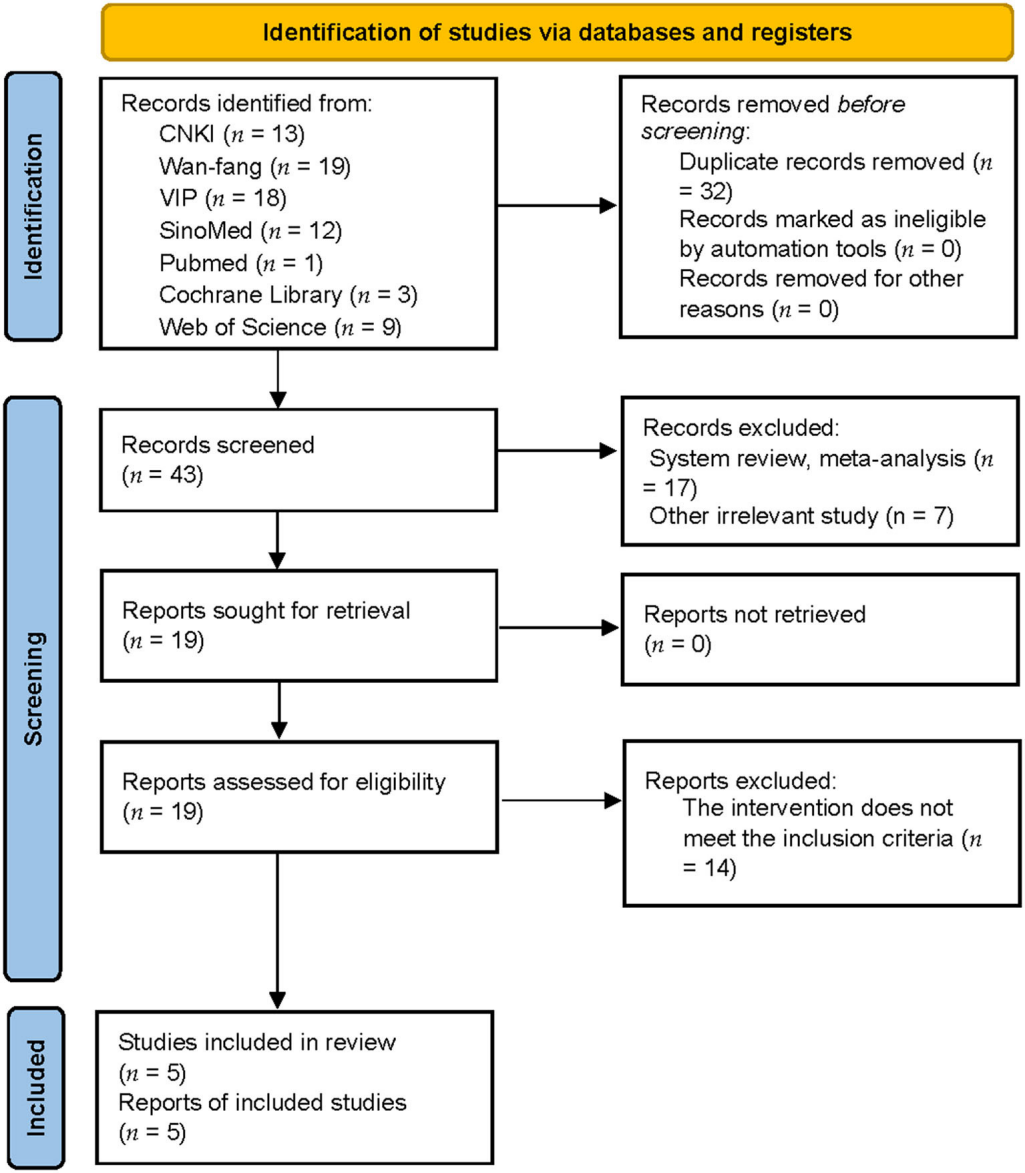
Flow diagram depicting the study selection process (n, number of publications).

A total of 75 publications were identified (13 articles from CNKI, 19 articles from the Wan-fang database, 18 articles from VIP, 12 articles from SinoMed, 1 article from PubMed, 3 articles from Cochrane Library, and 9 articles from Web of Science); these were imported into Endnote X9 (Clarivate Analytics). After eliminating duplicates, 43 articles were retained. Reviews and other irrelevant studies were excluded, thus leaving 19 studies. Studies featuring mixed interventions were also excluded. Finally, five trials remained for analysis after reading their full texts. Figure 1 shows a detailed flowchart of the study selection process.

### Characteristics of the Included Studies

A total of five articles were included in our analysis; these involved 270 patients with dysphagia after stroke (135 patients in the control group and 135 patients in the experimental group). The intervention in the control group was routine treatment, while that in the experimental group was routine treatment combined with mirror therapy. The first article was published in 2015. A total of two trials recruited convalescent patients recovering from stroke and three trials recruited patients in the acute phase. The shortest interventional period was 2 weeks; the longest was 8 weeks. With regard to the outcome measures, five trials reported the clinical effectiveness rate and water swallowing test whereas three trials reported the incidence of pneumonia. Table 1 shows detailed characteristics related to the included studies.

**Table 1:** Characteristics of the studies included in this meta-analysis.

**Study**	**Publish year**	**Sample**		**Intervention**		**Age (years)**		**Sex (M/F)**		**Stroke onset**		**MT protocol**	**Outcome measure**
		**C**	**E**	**C**	**E**	**C**	**E**	**C**	**E**	**C**	**E**		
Zhang (33)	2016	30	30	RT+ Sham MT (video of landscapes)	RT+MT	57.42 ± 2.65	57.30 ± 2.30	17/13	18/12	7.77 ± 1.27	7.81 ± 1.23	Video of swallowing, duration of the video was 5 min 16 s, two times per day, 6 days a week for 4 weeks.	WST, CER, the incidence of pneumonia
Long et al. (34)	2015	30	30	RT	RT+MT	56.61 ± 2.64	56.32 ± 2.32	18/12	19/11	8.14 ± 1.17	8.11 ± 1.13	Video of swallowing, duration of the video was 7 min, three times per day for 4 weeks.	WST, CER, the incidence of pneumonia
Li et al. (35)	2019	25	25	RT	RT+MT	50.27 ± 10.89	48.84 ± 11.62	16/9	18/7	50.71 ± 18.04	45.87 ± 16.93	Video of swallowing, duration of the video was 15 min, two times per day, 5 days a week for 8 weeks.	WST, CER, the incidence of pneumonia
Ju et al. (36)	2019	30	30	RT	RT+MT	61.3 ± 9.46	65.4 ± 8.95	17/13	18/12	6.67 ± 1.23	6.53 ± 0.92	Video of swallowing, duration of the video was 30–35 min, two times per day for 4 weeks.	WST, CER
Guan et al. (37)	2021	20	20	RT	RT+MT	43.15 ± 7.31	42.21 ± 8.42	11/9	12/8	31.96 ± 16.38	32.74 ± 15.57	Video of swallowing, duration of the video was 13 min, two times per day for 8 weeks.	WST, CER

*C, control group; E, experimental group; RT, routine treatment; MT, mirror therapy; WST, water swallowing test; CER, clinical effectiveness rate*.

### Risk of Bias Assessment

**Figure 2:**
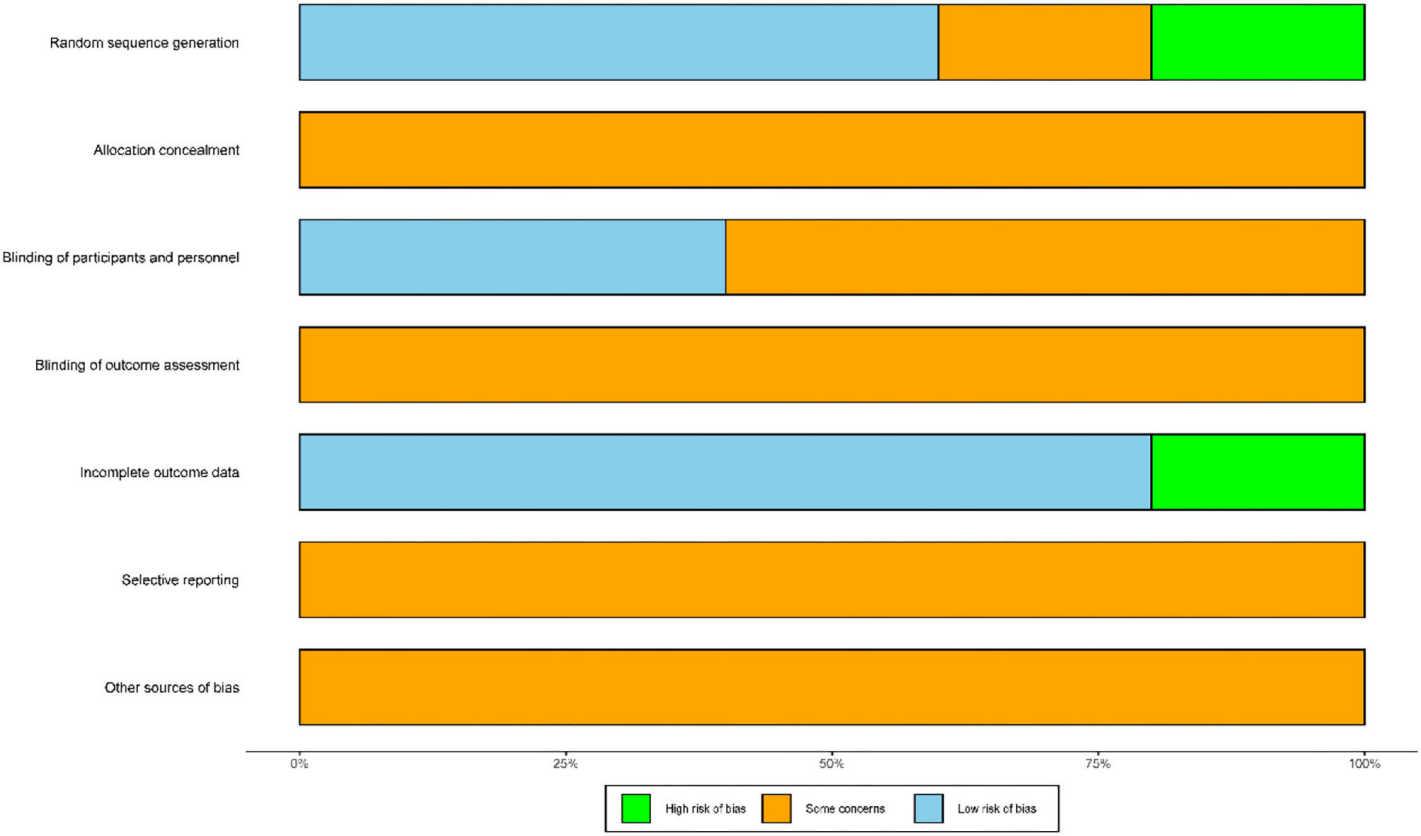
Risk of bias assessment.

Figure 2 presents a summary of the risk of bias associated with the included studies. In terms of random sequence generation, three trials reported that they had used random sequence generation and were determined to be low risk (34–36). Only one trial recruited subjects based on the hospital admission sequence and was considered as high risk (33). Then, one other study only mentioned the word “random” and was considered as unclear risk (37). With regard to allocation concealment, none of trials described the methods used for allocation concealment and were associated with an unclear risk. With regard to the blinding of participants and personnel, two trials used sham mirror therapy and were considered as low risk (33, 37), whereas three trials did not use sham mirror therapy and were classified as unclear risk (34–36). A total of five trials did not mention the method of blinding used for outcome assessment and were considered as unclear risk (33–37). A total of four trials reported complete datasets and were considered as low risk (34–37), whereas one trial featured an incomplete dataset and was considered high risk (33). None of the trials were clear with regard to selective reporting and other sources of bias and were thus classified as unclear risk (33–37).

### Results of the Meta-Analysis

#### Clinical Effectiveness Rate

**Figure 3:**
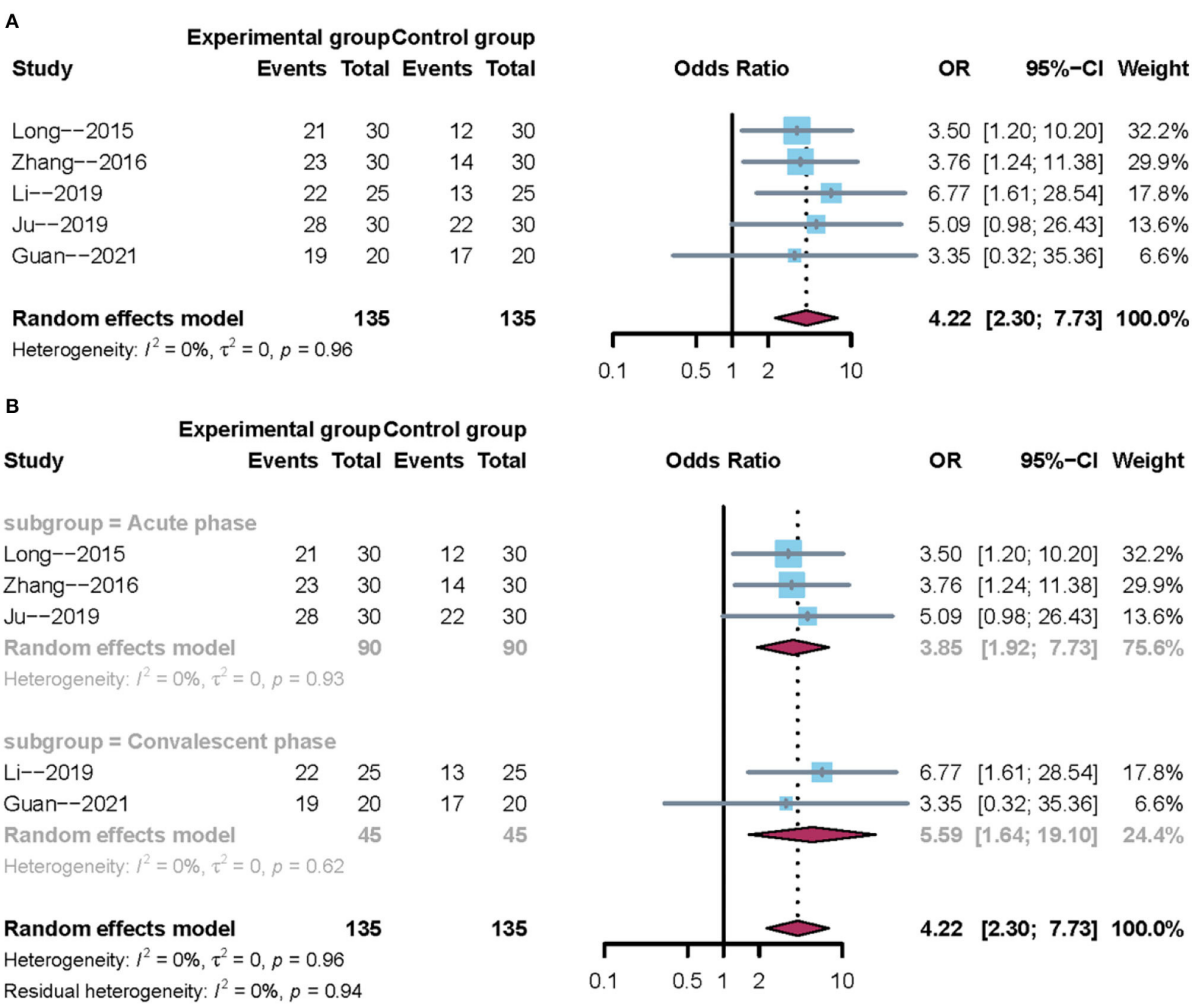
Forest plot of the clinical effectiveness rate. **(A)** Overall analysis of five included trials. **(B)** Subgroup analysis on the stroke phase (Acute phase vs. Convalescent phase).

A total of five trials reported the clinical effectiveness rate. After carefully reading the full text of the included studies, it was evident that the course of stroke differed across the five trails. Hence, subgroup analysis was performed according to the course of disease. Owing to clinical heterogeneity and potential methodological heterogeneity, even the *I*^2^ statistic <50%, the random-effects model was used to perform meta-analysis. There was a significant difference between mirror therapy and non-mirror therapy with regard to the clinical effectiveness rate (OR = 4.22; 95% CIs = 2.30–7.73), as presented in Figure 3A. Subgroup analysis showed that the convalescent phase had a larger positive effect size than the acute phase on post-stroke dysphagia (Figure 3B). In addition, sensitivity analysis showed that the results of this meta-analysis were stable.

#### Water Swallowing Test

**Figure 4:**
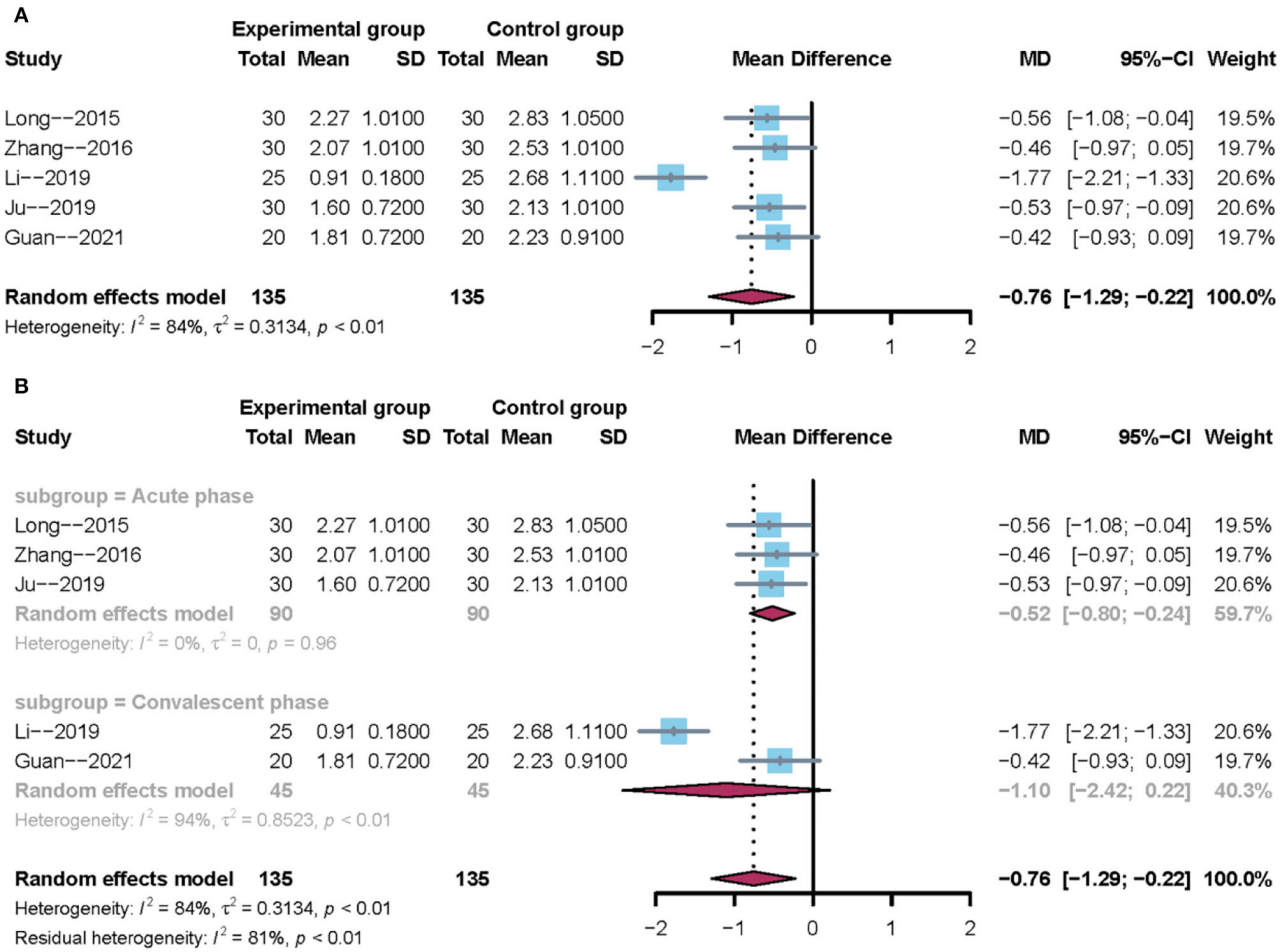
Forest plot of the water swallowing test. **(A)** Overall analysis of five included trials. **(B)** Subgroup analysis on the stroke phase (Acute phase vs. Convalescent phase).

All trials reported the water swallowing test. Owing to clinical heterogeneity and potential methodological heterogeneity, we used a random-effects model regardless of the level of heterogeneity. The results showed that there was no significant difference between mirror therapy and non-mirror therapy with regard to the water swallowing test (MD = −0.76; 95% CI = −1.29 to −0.22), as presented in Figure 4A. Subgroup analysis further showed that mirror therapy in the acute phase had a positive effect size (–MD = 0.52; 95% CI = −0.80 −0.24). However, mirror therapy during the convalescent phase had no positive effect size (MD = −1.10; 95% CI: −2.42 to 0.22), as shown in Figure 4B. In addition, sensitivity analysis showed that the results of this meta-analysis were stable.

#### The Incidence of Pneumonia

**Figure 5:**
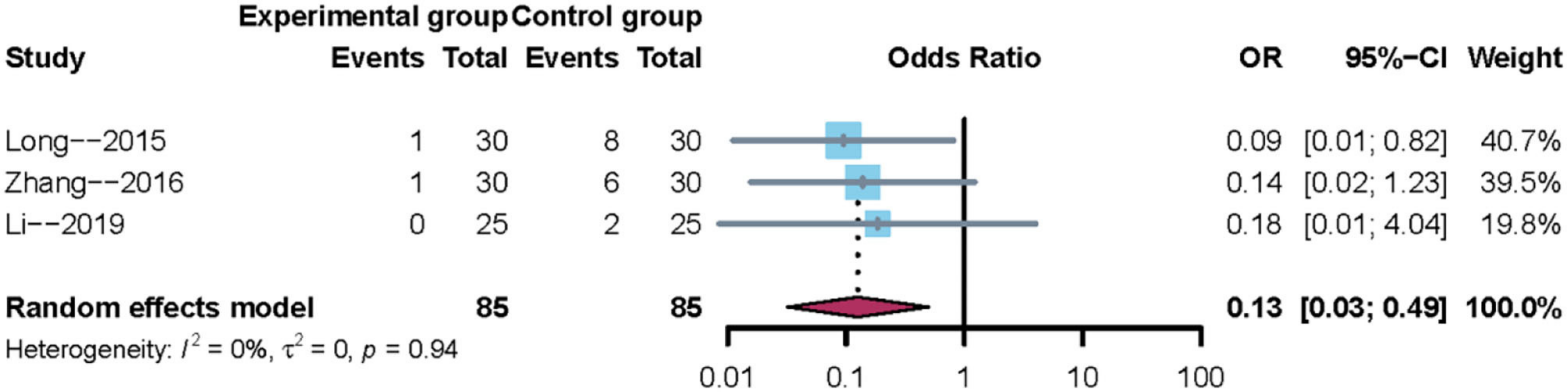
Meta-analysis of the incidence of pneumonia.

A total of three trials reported the incidence of pneumonia. Owing to clinical heterogeneity and potential methodological heterogeneity, even the *I*^2^ statistic <50%, the random-effects model was used to perform meta-analysis. The results showed that there was a significant difference between mirror therapy and non-mirror therapy with regard to the incidence of pneumonia (OR = 0.13; 95% CI = 0.03–0.49), as shown in Figure 5. In addition, sensitivity analysis showed that the results of this meta-analysis were credible.

#### Publication Bias

**Figure 6:**
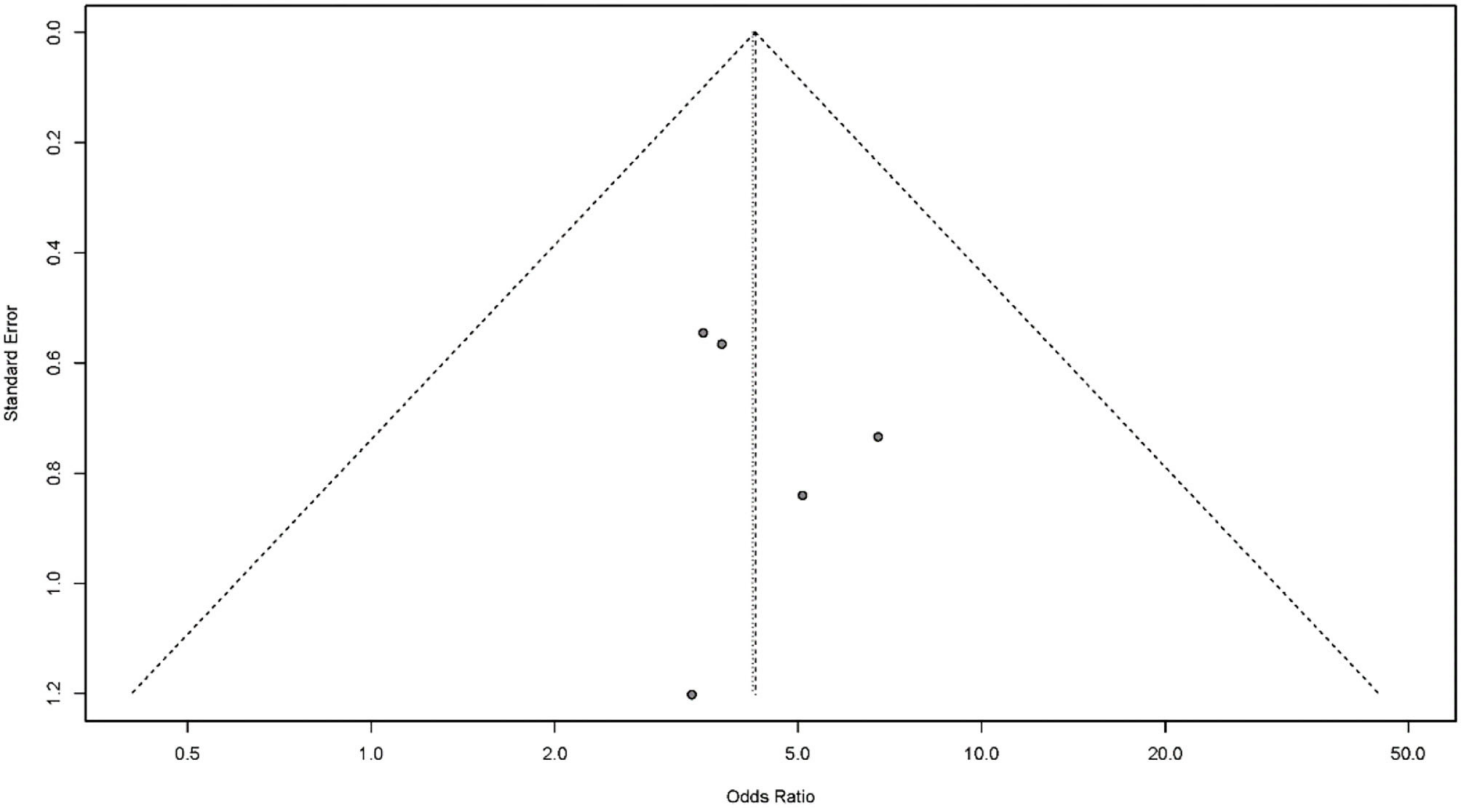
Funnel plots for the assessment of publication bias.

Publication bias is a potential concern when interpreting the results of meta-analysis. Here, we used a funnel plot and Egger's bias indicator test to assess publication bias in the five studies included in our final analysis. Publication bias was indicated by an asymmetric funnel around the pooled effect size. It is worth noting that the five studies lay symmetrically around the pooled effect size. Egger's bias indicator test also confirmed that there was no evidence of publication bias (*p* > 0.05; Figure 6).

## Discussion

The results of this study indicated that mirror therapy may play a positive role in treating post-stroke dysphagia. According to subgroup analysis, mirror therapy during the acute and convalescent phases after a stroke was associated with a positive effect size in terms of clinical effectiveness rate. In terms of the water swallowing test, mirror therapy during the acute phase of stroke showed a positive effect size. However, mirror therapy during the convalescent phase of stroke had no positive effect size. Sensitivity analysis showed that this evidence was stable. In addition, there was no evidence of any publication bias. To the best of our knowledge, this is the first study to investigate the efficacy and safety of mirror therapy on post-stroke dysphagia. Consequently, our results are valuable and important for both the patient and clinic.

Due to the lack of a systematic review or meta-analysis focused on the synergic effects of mirror therapy, our present findings were compared to similar, but not specific, research studies. Most previous studies focused on the hand-mirror system (38). In addition to the activation of corresponding mirror neurons by hand movements, mirror neurons can also respond to the stimulation of mouth movement and become active when individuals perform or observe lip activities related to feeding function (39). In addition, the firing of mirror neurons was temporally and closely associated with grabbing, lip movement, crushing, or sucking (40). A previous study using functional magnetic resonance imaging showed that the visual and audiovisual stimuli associated with swallowing motion can activate cortical areas related to the swallowing motor, such as the auxiliary motor area, premotor area, primary motor area, and cingulate gyrus (41). Another previous study used magnetoencephalography to detect a positive relationship between the activated regions of the cerebral cortex and the mirror neuron system during swallowing (21). Based on the existing evidence, we were able to provide answers to key research questions, especially with regard to swallowing function and the incidence of pneumonia. The positive results for clinical effectiveness rate were consistent with those for the water swallowing test. To a certain extent, the positive effects identified here suggest that mirror therapy as a non-invasive therapeutic and rehabilitative intervention can improve the swallowing ability of patients after stroke. However, these results are limited because our subgroup analysis indicated that the treatment effects between the acute and convalescent phases of stroke were not uniform.

We found that the use of mirror therapy to treat swallowing function has a positive effect size during the acute phase of stroke but not during the convalescent phase. This difference may be related to several factors.

First, it is known that mirror therapy can be applied to different phases of patients with stroke (acute, subacute and chronic stroke) (42–44). One previous study reported that early intervention was promising with regard to the treatment of swallowing function after stroke (45). This might explain the different treatment effects between the acute and convalescent phases of stroke. It is possible that the inconsistencies between the pooled and subgroup analyses may be attributable to significant heterogeneity between subgroups. Moreover, it is possible that the subgroup analysis may not be reliable because only a small number of trials were included in each subgroup and that multiple subgroup analyses can lead to spurious results. Further research is required to investigate these factors further. Despite the detection of positive results, several problems are evident in the five studies analyzed here. Although mirror therapy is extensively used to treat post-stroke dysphagia in clinical practice, the application of mirror therapy still remains limited. All the trials were performed in China; no similar work has been reported for the treatment of post-stroke dysphagia in any other country. Because specific stroke subtypes were not specified the studies included here, these results cannot be universally applied, even in the Chinese population. In addition, there are no unified criteria for applying mirror therapy at present (18, 46). The implementation of treatment schemes (such as video content setting, observation and execution time, duration of treatment, and evaluation indicators) that were described across different publications is known to vary in clinical practice. In addition, mirror therapy is a therapeutic option based on the integration of observation, imagination, and imitation; thus, the strategy relies upon the cognition and vision of the patients being tested, thus limiting the scope of application.

Second, we generated funnel plots and performed Egger's test for publication bias (32). To our surprise, Egger's test for publication bias was not significant (*p* > 0.05), thus implying that the five studies included in our analysis were unlikely to feature significant publication bias. Even though Egger's test suggested that there was no publication bias, the limitations imposed by potential publication bias should be considered because it is easier to report positive results (47, 48). Furthermore, we should not ignore the fact that most studies evaluating patient-reported outcomes did not use a placebo-controlled design. Placebos serve as a control to discriminate the effects of an active treatment from the non-specific components of the treatment (49). With regard to placebo, expectancy is usually defined as the subjective probability of the occurrence of a specific clinical outcome (50). However, only one study included used sham mirror therapy (33). As a result, we cannot rule out the potential confounding effect of placebo on our findings.

Finally, all of the assessment scales used in this study were indirect assessment methods and therefore lacked direct observation methods and imaging detection; this may have had an effect when determining the specific impact on the effective rate of treatment. It is well-known that the gold standard methods for evaluating swallowing functions are flexible endoscopic evaluation of swallowing (FEES) and video-fluoroscopic swallowing studies (VFSS) (51, 52). However, no datasets are available yet for these methods. Thus, potential clinical heterogeneity could not be excluded. Therefore, we cannot draw definite conclusions based on the results from the studies analyzed herein.

## Study Limitations

Several specific limitations need to be considered with regard to this study. First, the currently available evidence related to the use of mirror therapy for post-stroke dysphagia originated from a relatively sample size; furthermore, there were limited numbers of subjects in each trial and limited analysis of outcome data. Therefore, there is a need to carry out further studies with a larger sample size using more objective indicators. Second, safety indicators may not be optimal and follow-up data are lacking. Hence, we could not fully evaluate the safety profile of mirror therapy or the long-term efficacy of this technique in the target patient population. Third, although we performed specific searches in a wide range of databases, all of the included trials were performed in China; as such, our results may not be representative on a wider scale.

## Conclusion

Mirror therapy may have a positive role in the management of post-stroke dysphagia but has yet to be specifically confirmed. In addition, data from existing trials suggest that evidence for the safety of mirror therapy in patients with post-stroke dysphagia is not yet sufficient.

## Data Availability Statement

The original contributions presented in the study are included in the article/supplementary materials, further inquiries can be directed to the corresponding author/s.

## Author Contributions

KH conceived the study and drafted the paper. LW and FN developed the search strategies. XL and KL performed literature selection and data extraction. RM conceived the study, designed the protocol, and assisted with data acquisition, analysis, and drafting the paper. All authors contributed to the article and approved the submitted version.

## Funding

This work was supported by the Chinese Medicine Research Program of Zhejiang Province (Nos. 2020ZX011, 2022ZQ047, and 2022ZB198), the Zhejiang Chinese Medical University Research Fund (No. 2018ZY17), and the Traditional Chinese Medicine of Zhejiang Province Science and Technology Plan Project (No. 2018ZQ027).

## Conflict of Interest

The authors declare that the research was conducted in the absence of any commercial or financial relationships that could be construed as a potential conflict of interest.

## Publisher's Note

All claims expressed in this article are solely those of the authors and do not necessarily represent those of their affiliated organizations, or those of the publisher, the editors and the reviewers. Any product that may be evaluated in this article, or claim that may be made by its manufacturer, is not guaranteed or endorsed by the publisher.
